# Nanoplastic Labelling with Metal Probes: Analytical Strategies for Their Sensitive Detection and Quantification by ICP Mass Spectrometry

**DOI:** 10.3390/molecules26237093

**Published:** 2021-11-24

**Authors:** Lucile Marigliano, Bruno Grassl, Joanna Szpunar, Stéphanie Reynaud, Javier Jiménez-Lamana

**Affiliations:** Institute of Analytical and Physical Chemistry for the Environment and Materials (IPREM), Universite de Pau et des Pays de l’Adour, E2S UPPA, CNRS, 64053 Pau, France; bruno.grassl@univ-pau.fr (B.G.); joanna.szpunar@univ-pau.fr (J.S.); stephanie.reynaud@univ-pau.fr (S.R.); j.jimenez-lamana@univ-pau.fr (J.J.-L.)

**Keywords:** ICP-MS, single particle, nanoplastics, metal labels, adsorption

## Abstract

The detection and quantification of nanoplastics in aquatic environments is one of the major challenges in environmental and analytical research nowadays. The use of common analytical techniques for this purpose is not only hampered by the size of nanoplastics, but also because they are mainly made of carbon. In addition, the expected concentrations in environmental samples are below the detection limit of the majority of analytical techniques. In this context, the great detection capabilities of Inductively Coupled Plasma Mass Spectrometry (ICP-MS) in its Single Particle mode (SP-ICP-MS) have made of this technique a good candidate for the analysis of nanoplastics. Since the monitoring of carbon by ICP-MS faces several difficulties, the use of metal tags, taking advantage of the great potential of nanoplastics to adsorb chemical compounds, has been proposed as an alternative. In this perspectives paper, three different strategies for the analysis of polystyrene (PS) nanoplastics by SP-ICP-MS based on the use of metals species (ions, hydrophobic organometallic compound, and nanoparticles) as tags are presented and discussed. Advantages and disadvantages of each strategy, which rely on the labelling process, are highlighted. The metal nanoparticles labelling strategy is shown as a promising tool for the detection and quantification of nanoplastics in aqueous matrices by SP-ICP-MS.

## 1. Introduction

Nanoplastics (NPTs) are plastic debris of mixed composition and shape with sizes below 1 µm resulting from the degradation of industrial plastics [1], potentially present in all environmental compartments. Indeed, million tons of plastics produced since last century end up in the ocean each year [2], where they gradually degrade into mesoplastics, and thereafter into microplastics (<5 mm) and NPTs through mechanical abrasion, photodegradation, thermooxidative degradation, hydrolysis, and biodegradation [3,4,5]. Once in the environment, NPTs are expected to be persistent and able to pass through biological barriers and be translocated to different organs in aquatic organisms [6]. In addition, due to their higher surface-to-volume ratio compared to microplastics they are expected to act as carriers of other pollutants (metals, pesticides, PCBs) in a much more efficient way than their bigger counterparts [7].

However, the investigation of the occurrence, transport, metabolism, and reactivity of NPTs is hampered by the lack of methods able to identify and quantify sub-microsized plastics in the environment. We are facing a prosaic challenge: because of their low size and of the fact that they are mainly made of carbon, they elude most of the instrumental techniques available and demand for novel ingenious analytical methodologies. The majority of the methods used for the analysis of microplastics cannot be directly applied for NPTs.

The development of methods able to characterize and quantify NPTs in the environment requires NPT model materials. The occurrence of NPTs in the environment mostly results from the degradation of plastic waste by different biological and/or abiotic processes i.e., thermodegradation, photodegradation, mechanical fragmentation, hydrolysis, and biodegradation [6] that can alter the properties of NPTs. Photo and thermo degradation are particularly important pathways as they lead to chemicals transformations through oxidative effects that enhance the surface functionalities of NPTs and hence, their ability to interact with other contaminants present in the environment [8,9]. In this context, NPTs studies must be done with model materials representative of degradation and transformations they can undergo in the environment. Commercially available monodisperse and spherical polystyrene latex particles (PSLs), certified in terms of size, have been so far mostly used in studies about the impact and the behaviour of NPTs. Nevertheless, those NPTs synthesized through a bottom-up approach may contain solvents, surfactants, preservative residues as sodium azide, trace metals, and other substances that may induce bias when measuring their physical, chemical, and biological behaviour. Furthermore, there is no or poor data concerning their surface functionality and surface morphology, which is crucial information when considering NPTs as vectors of contaminants. The study presented here has been performed with synthesized PS NPTs using a soap-free emulsion polymerization leading to NPTs with functionalized surface by carboxylic groups. These NPTs are monodisperse in size (PDI < 0.05), metal-free, and exhibited smooth or raspberry-like surface morphologies. The NPT samples are called as PSAAxx, from their chemical composition, i.e., PS for polystyrene and AAxx to notice that a monomer ratio xx = [AA]/[S] of acrylic acid over styrene is used to functionalize the surface.

Besides the studies that deal with the detection of NPTs [10,11], methods devoted for their quantification are still lacking. The determination of mass concentration is mostly performed using mass spectrometry techniques [12,13,14], making possible the simultaneous identification and quantification of NPTs present in the samples. In another study, the use of fluorescence-based methodology by using fluorescent molecular rotors probe enabled the mass concentration determination of NPTs in biological tissues [15]. On the other hand, the number concentration is often determined using Nanoparticle Tracking Analysis (NTA). This non-specific method allows simultaneously the determination of the size, size distribution and number concentration of NPTs down to 10^9^ part L^−1^. For now, NTA has never been reported for the quantification of NPTs in environmental samples. However, it has been used for the quantification of NPTs formed during fragmentation process of larger plastics [16,17,18,19]. The determination of number concentration of NPTs has also been reported using scanning electron microscopy (SEM) [20,21]. Techniques based on electron microscopy might appear as convenient tools to determine the number concentration of NPTs. Nonetheless, they are limited by the sample preparation, which lead to low recoveries due to sample preparation [22,23]. Recently, Molenaar et al. proposed the quantification of NPTs by fluorescence microscopy combined to single particle tracking (SPT) using NileRed for the labelling of PS nanoplastic standards [24]. Through this technique, a particle diameter as low as 45 nm with a limit of detection of 2 × 10^9^ part L^−1^ was determined.

According to our knowledge, no method is currently capable of quantifying NPTs present in the environment. This is mainly due to the fact that the low concentrations of NPTs in the environment are below the detection limits of majority of analytical instruments. Based on the extrapolation proposed by Lenz et al. [25], Reynaud et al. estimated the concentration of nanoplastics with a hydrodynamic size lower than 1 µm at 0.14 to 1.4 ng L^−1^ in our oceans, which represents about 0.1 to 1% of the 200 Mt of plastic waste already accumulated in the environment [26]. In this context, the use of ICP-MS in single particle mode may sound as an attractive alternative since it has become a standard technique for the analysis of engineered nanoparticles (ENPs) [27]. SP-ICP-MS allows for the determination of the particle concentration as well as the size and the agglomeration state of the nanoparticles, on very dilute suspensions, i.e., in the range of µg L^−1^ and down to ng L^−1^. However, the analysis of carbon-based nanomaterials by SP-ICP-MS technique is hampered by the low sensitivity of carbon towards ICP-MS (mainly because of its low ionization efficiency and high ionization potential) and by the presence of carbon in water and air, which results in a high background when monitoring the most abundant isotope of carbon (^12^C). Recently, some authors have attempted the analysis of microplastics through the monitoring of ^13^C [28,29]. However, the detection of plastics debris with sizes below 1 µm was not possible due to a lack of sensitivity.

In this perspectives paper, the labelling of NPTs with a metallic compound, through hydrophobic interactions, π-π-interactions, and electrostatic interactions, which renders them detectable to SP-ICP-MS, is proposed. The use of three different metal probes (metal ions, hydrophobic organometallic compound, and inorganic nanoparticles) is discussed and their advantages and disadvantages highlighted.

## 2. Labelling Strategies

### 2.1. Labelling with Metal Ions

It has been shown that small plastic debris is able to adsorb trace metals in the aquatic environment [30]. The labelling of NPTs with metal ions is expected to take place through electrostatic interactions. In this scenario, the adsorption is based on chemical interactions between the carboxylate groups of the functionalized surface of NPTs and the positive metal ions in solution. The first step was to select a suitable element for the labelling of NPTs with metal ions. One of the preliminary conditions is that the metal probes must remain stable in solution at the working conditions to avoid any precipitation or formation of oxides.

**Figure 1:**
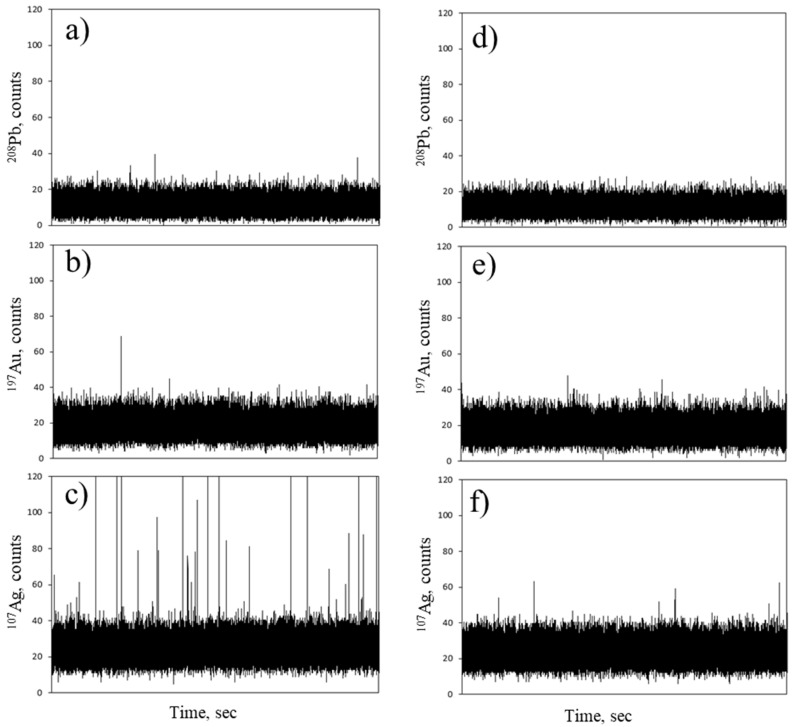
Time scans obtained by SP-ICP-MS for a water dispersed PSAA13 nanoplastic model material mixed with (**a**) ionic Pb; (**b**) ionic Au; (**c**) ionic Ag and for ionic solutions of (**d**) Pb, (**e**) Au, (**f**) Ag at the same concentration without nanoplastics.

A preliminary test was carried out using gold, lead, and silver ionic solutions for the labelling of PSAA13 model material. A sample of each metal probe was prepared, containing final concentrations of 50 µg L^−1^ and 1 µg L^−1^ of the corresponding metal ion and NPTs, respectively. The expected NPT number concentration in the sample was around 2.7 × 10^9^ L^−1^. Figure 1 shows the time scans obtained in SP-ICP-MS. No significant differences between the time scan obtained for ionic metal solution (gold or lead) with and without NPTs were visible, suggesting that the sorption does not occur, or that the amount of metal adsorbed is not high enough to produce a signal that can be distinguished from the background. In contrast, the time scan obtained for the NPTs labelling with silver showed a significant number of peaks compared to the ionic solution without NPTs, suggesting that enough silver atoms were adsorbed onto the NPTs in order to render them visible to the ICP-MS detector.

However, the number of peaks detected was not significant for quantification purposes under these conditions. In addition, the background contribution, mostly due to free silver that remains in solution at the equilibrium (i.e., not bound to NPTs) is predominant. Hence, the feasibility of metal ionic sorption on NPTs using ionic silver was first assessed using an adsorption isotherm. The NPT model material PSAA22, with a higher surface functionalization, was chosen, and AgNO_3_ salt was used for the preparation of ionic solutions. Mixtures of PSAA22 at 100 mg L^−1^ with different silver concentrations (Table 1) were prepared.

**Figure 2:**
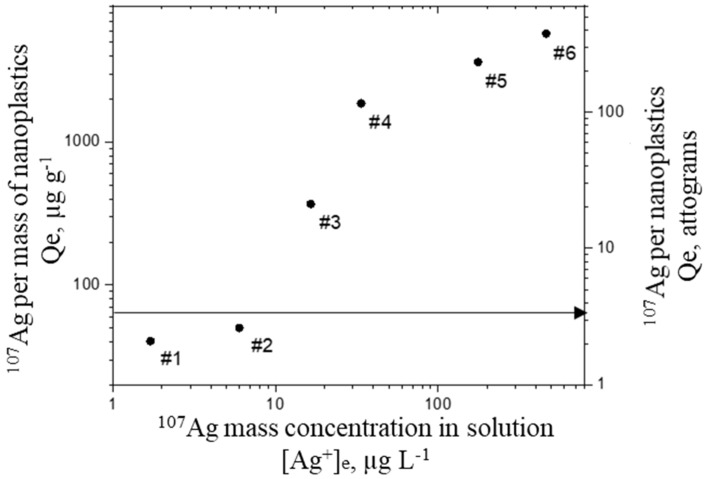
Mass of silver adsorbed onto nanoplastics with respect to the free silver ionic concentration at the equilibrium in different mixtures PSAA22:Ag. The arrow indicates the minimum mass of silver required per nanoplastic for SP-ICP-MS detection.

Through this approach, the amount of metal adsorbed on NPTs is indirectly calculated through the determination of the silver concentration that remains in the filtrated solution. The mass of silver adsorbed per mass of plastic and per NPT particle (Qe) as a function of the equilibrium concentration ([Ag^+^]_e_) that remains in the solution are shown in Figure 2.

The results clearly show that the adsorption of silver onto NPTs occurs, as the quantity of silver retained by NPTs (Qe) increases with the initial concentration of silver. For the experiment with the highest amount of silver spiked (i.e., #6), the mass of metal adsorbed per NPT reached 355 attograms (ag). With the assumption that one carboxylate group binds on one silver atom, this amount of silver represents 10% of the total amount of carboxylic groups present on the NPT surface. The minimum mass required to be adsorbed onto each NPT for their detection by SP-ICP-MS can be calculated through the intensity critical value (*I_C_*), defined as the response of the instrument above, which an observed signal is reliably attributed to the presence of a particle [31], using the following equation:

(1) $$m_{min}=\frac{I_{C}\;}{K}\times t_{d}\times q\times \eta$$

where *m_min_* is the minimum mass adsorbed per NPT, *K* the response factor of the instrument to the corresponding isotope, *t_d_* the dwell time, *q* the sample uptake flow, and *η* the transport efficiency. For the best-case scenario (i.e., for a background signal between 0 and 1), this value was calculated as 3.68 ag part^−1^ in the case of silver. Consequently, the detection and quantification of PSAA22 NPTs by SP-ICP-MS remains not feasible for the two experiments with the lowest amount of metal (#1 and #2, Table 1), which gave 2.5 and 3 ag part^−1^, respectively.

**Figure 3:**
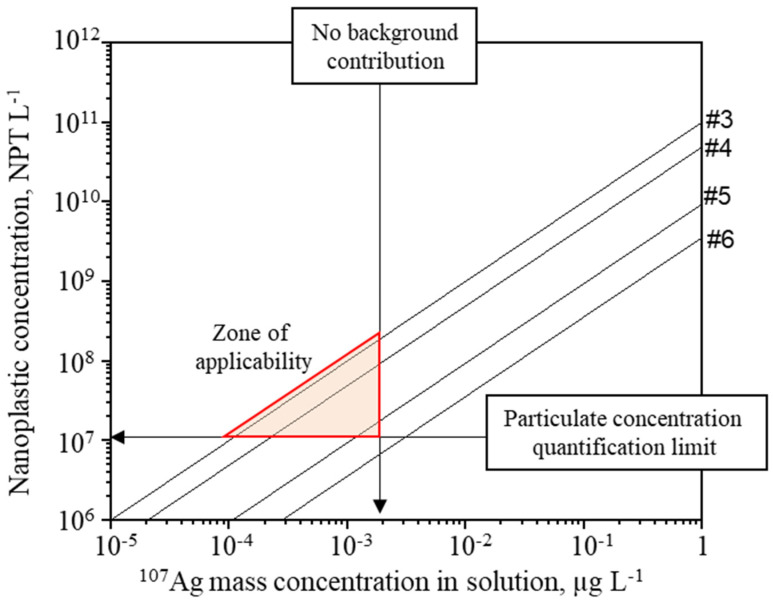
Theoretical silver concentration for the different experiments if no modification of Qe occurs.

Since the initial concentration of PS22AA in the experiments (around 1.62 × 10^12^ NPT L^−1^) is too high for Single Particle analysis [32], samples must be diluted in order to ensure that only one NPT is detected at each reading time. However, the NPTs concentration must remain above a certain limit for its accurate quantification by SP-ICP-MS. For inorganic nanoparticles, LOD obtained by SP-ICP-MS were reported to be in the range of 10^5^–10^6^ part L^−1^ [33], thus a threshold of 10^7^ part L^−1^ was selected here for a proper quantification of NPTs in the aim of further analytical development. On the other hand, concerning the background signal, the real situation is far from the best-case scenario mentioned above, and hence the analysed water dispersions must be diluted enough to minimize the impact of the free silver that remains in solution. The expected concentration of free silver in the diluted sample can be calculated using the value [Ag^+^]_e_ obtained during the adsorption isotherm experiment, assuming that no modification of the adsorbed quantity occurs during the dilution. Figure 3 presents the expected ionic concentration of free silver in the different samples analysed following the NPTs concentration in the diluted samples with the assumption that no modification of Qe occurs. Under such conditions, the quantification of NPTs was not feasible in samples #5 and #6 due to the expected high amount of free silver in the suspension. Nonetheless, a narrow zone of applicability for samples #3 and #4 can be considered.

Hence, 20,000-fold and 1000-fold dilutions were applied to sample #4, which corresponded to 8 × 10^7^ NPT L^−1^ and 1.6 × 10^9^ NPT L^−1^, respectively, and were analysed by SP-ICP-MS (Figure S1). The total concentration of silver represented 10 ng L^−1^ for the 20,000-fold dilution suspension. Hence, NPT number concentration and ionic background in this suspension should be then adequate for SP-ICP-MS analysis. Results showed that no ionic contribution were detected; however, no pulses were detected either on the time scan (Figure S1a). This result suggests that NPTs did not have enough silver adsorbed for their detection, which can be explained by a desorption process during dilution. For the 1000-fold dilution suspension, the total concentration of silver in the sample represented 200 ng L^−1^ with an expected free silver ionic concentration of 30 ng L^−1^. The time scan obtained by SP-ICP-MS analysis showed that the number of peaks detected were not significant for quantification purposes (Figure S1b). On the other hand, since results revealed a free silver ionic concentration of 160 ng L^−1^, the hypothesis of desorption cannot be excluded.

In conclusion, the detection of NPTs by SP-ICP-MS through metal ions labelling is hampered by different factors. First, the number concentration of NPTs required to be above 1 × 10^7^ NPT L^−1^ for quantification in an analytical development perspective. Second, the mass of metal adsorbed onto NPTs needs to be sufficient to be detected by SP-ICP-MS. Finally, the ionic background needs to be low enough to not hide NPTs’ signal contribution. In addition, desorption can occur during dilution process, which can be explained by an equilibrium process. The present study does not assert the unfeasibility of the metal labelling, however, these results show the complexity of this strategy and the necessity to investigate other labelling strategies.

### 2.2. Labelling with a Hydrophobic Organometallic Compound

Plastic materials have been reported to have high sorption capacity with hydrophobic organic chemicals (HOCs) [34]. Therefore, the labelling of NPTs was performed by taking advantage of the hydrophobic interactions that occur between their surface and HOCs [35]. Lead (II) phthalocyanine (PbPc) is an aromatic hydrophobic organic compound chosen for its hydrophobicity and its capability to be quantified through SP-ICP-MS by monitoring the lead included inside the compound.

**Figure 4:**
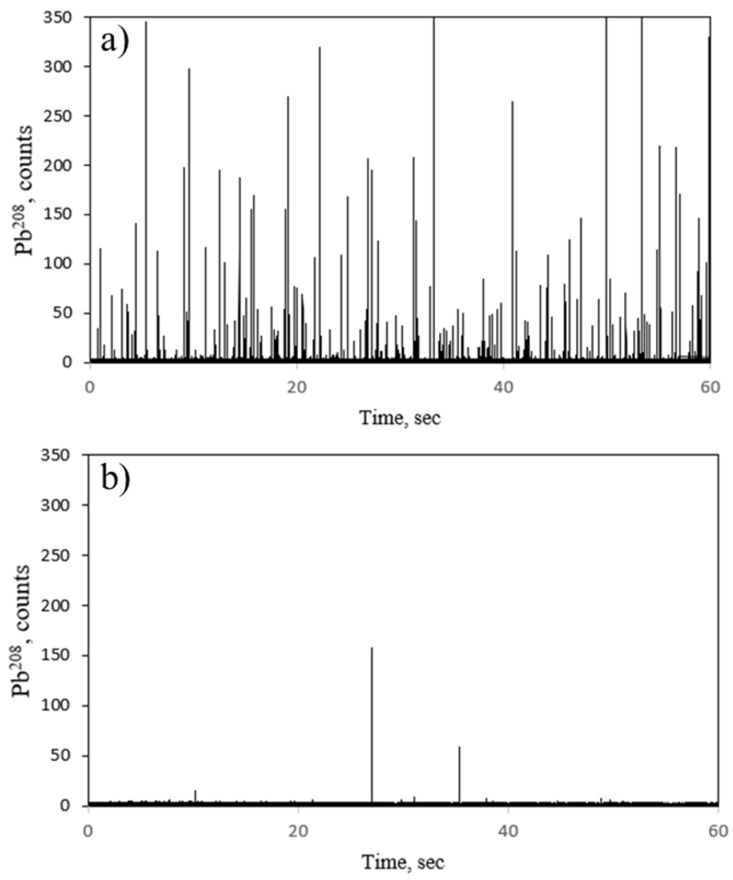
(**a**) Mixture of PSAA22 and Lead (II) phthalocyanine (**b**) Lead phthalocyanine in water in the absence of PSAA22; after centrifugation.

The labelling of PcPb on PSAA22 NPTs was carried out at a mix ratio PSAA22:PcPb of 500:0.00025 in mg L^−1^ corresponding to an expected NPT concentration of 2.83 × 10^13^ NPTs L^−1^. The choice of the solvent was done through a compromise between the pH and solubility of the product. For instance, PbPc was shown to be soluble in organic acids like formic acid, but the resulting pH was not compatible with the NPTs suspension. In order to work with well-dispersed suspensions of NPTs, ultrapure water was chosen as matrix. Due to its hydrophobicity, the mixture of PbPc with NPTs in ultrapure water resulted in an equilibrium between the insoluble PbPc, precipitating as residual large green aggregates, visible to the naked eye, and the PbPc adsorbed on the water dispersed NPTs. As a result, centrifugation and filtration steps were performed in order to isolate NPTs from the insoluble aggregates (See Section 3). A 100,000-fold dilution was applied to the resulting purified fraction before SP-ICP-MS analysis. A significant number of pulses was observed in the time scan obtained by SP-ICP-MS for the labelled NPTs (Figure 4a), showing that a non-negligible fraction of PcPb became accessible to NPTs. In order to ensure that the signal observed came from labelled NPTs with PbPc, a method blank consisting of a sample of PcPb prepared following the same protocol method and at the same PcPb concentration, was analysed by SP-ICP-MS. The time scan obtained (Figure 4b) confirms that no false signals were obtained due to PcPb solution in the mixture as peaks above the baseline are negligible compared to the time scan obtained for labelled NPTs. The strong adsorption was explained by hydrophobic interactions and π-π-interactions of the monomer unit [36]. In terms of quantification, a 50% recovery in terms of nanoparticle number concentration was obtained by SP-ICP-MS, which suggests that the centrifugation and filtration steps entailed losses of labelled NPTs. In contrast to the metal labelling, the sorption efficiency of PcPb was such that the contribution of the free ionic PcPb was negligible for the quantification of NPTs. In conclusion, the use of an organometallic compound like lead (II) phthalocyanine as a metal probe is a good and simple strategy for the detection of NPTs in an aqueous medium, but it may lead to an underestimation of NPTs number concentration. In order to avoid centrifugation and filtration steps, this method requires investigations concerning adequate solvent able to solubilise PcPb while maintaining stability of NPTs dispersity. Alternatively, Hildebrandt et al. recently proposed continuous flow centrifugation, which could constitute another approach for the separation of nanoplastic from micrometer-sized suspended particulate [37].

### 2.3. Labelling with Metal Nanoparticles

Gold nanoparticles of 17 nm were chosen as particle models to study the feasibility of nanoplastic labelling. They are easy to elaborate and their low occurrence in the environment avoids the generation of background noise that could alter the analysis of environmental samples at a later stage.

The new strategy here consisted on labelling the NPT model materials by using custom-designed amine functionalized gold nanoparticles (AuNPs@gel). In this approach, metal nanoparticles labelling was achieved by the coupling of negatively charged carboxylate groups present at the surface of the NPT model materials with a positively amino charged gelatin attached to the custom-synthesized AuNPs.

**Figure 5:**
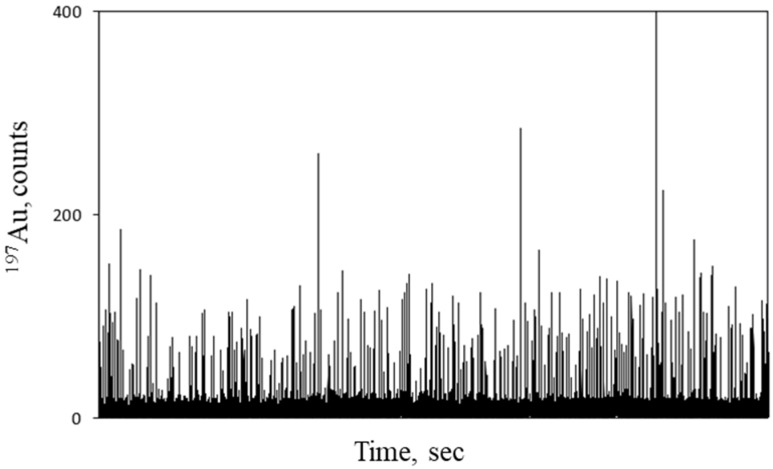
SP-ICP-MS time scan for the sample obtained for the mixture of PSAA22 and AuNPs@gel.

The feasibility of the method was tested onto the following series of NPT model materials: PSAA9, PSAA13, PSAA18, and PSAA22, which mainly differ in their functionalization of 2, 7, 43, and 57 carboxylic groups per nm^2^, respectively. Each NPT model material was mixed with a suspension of AuNPs@gel at a ratio of 1:1000 in terms of number of particles. The resulting mixture was diluted accordingly with water in order to have a NPT number concentration of around 1 × 10^8^ L^−1^ prior to SP-ICP-MS analysis. The time scans obtained by monitoring the ^197^Au signal showed a significant number of pulses above the background (Figure 5) that are due to AuNPs@gel attached to a single NPT. The background is suggested to be due to AuNPs@gel that are not linked to any NPT carriers and hence remain ‘free’ in the sample.

**Figure 6:**
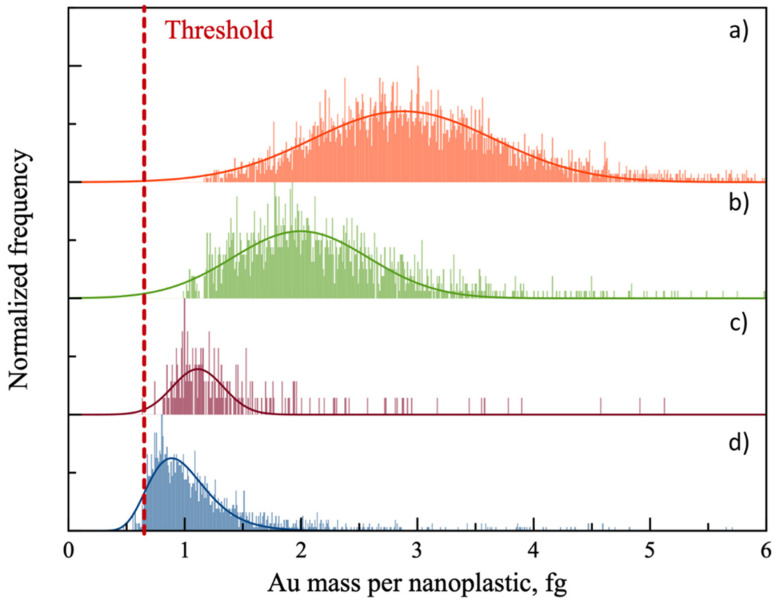
Au mass distributions obtained for samples made of a mixture of (**a**) PSAA22; (**b**) PSAA18; (**c**) PSAA13 and (**d**) PSAA9 nanoplastic model materials with AuNPs@gel. The red line represents the threshold discriminating between labelled nanoplastics and not bound AuNPs@gel in the sample.

The mass distributions obtained for the four NPTs model materials labelled with AuNPs@gel (Figure 6) revealed that the mass of gold adsorbed onto each NPT increased with the number of carboxylic groups per nm^2^ at their surface, confirming the electrostatic interactions mechanism. However, for PSAA9 and PSAA13 there is an overlap between the distribution due to the labelled NPTs and the distribution due to AuNPs@gel that are not bound to NPTs, which hampers the quantification of low functionalized NPTs. As a consequence, a critical value (or threshold) must be applied in order to isolate the former one. The mass distributions reported in Figure 6 showed the results obtained after applying a restrictive criterion of 5σ [31]. Although this criterion value differs from sample to sample, the threshold represented in Figure 6 is set at the same place for the 4 samples for the sake of illustrative purposes. This is confirmed by the NPT number concentrations obtained by SP-ICP-MS for the different samples: for the highest functionalized NPTs, PSAA22 and PSAA18, NPTs number concentrations in good agreement with expected values were obtained (Table 2). These results prove that the quantification of functionalized NPTs through metal nanoparticle labelling and SP-ICP-MS analysis is feasible. However, in the case of PSAA13 and PSAA9, the number concentration values obtained were around 20 times lower than the expected one, which suggests that part of those NPTs did not adsorb enough AuNPs@gel to be properly detected. Hence, the quantification of lower functionalized NPTs requires the adsorption efficiency to improve and/or the removal of the non-adsorbed AuNPs@gel. In order to promote the sorption of AuNPs@gel onto NPTs, some parameters should be investigated. For instance, the pH of the resulting mixture represents a pivotal parameter since the labelling of nanoplastics through electrostatic interactions using AuNPs@gel requires negatively charged nanoplastics (i.e., carboxylated) and positively charged gold nanoparticles. The literature reports pKa of 4.1 and 4.5, respectively, for the acrylic acid monomer and the poly(acrylic acid) [38,39]. As a consequence, at a pH of 5.5, carboxylic groups are overwhelmingly deprotonated. On the other hand, a pH below the isoelectric point of gelatine (≈8) ensured protonated groups at AuNPs@gel surface. Additionally, the ratio between NPTs and AuNPs@gel could strongly influence the amount of AuNPs@gel sorbed onto nanoplastic surface as well as background contribution due to equilibrium processes. Other parameters such as the the exposure time of the resulting mixture, or the size of AuNPs@gel used should be investigated to determine the optimal sorption conditions.

In a previous work, the applicability of the labelling of NPTs using gold nanoparticles by means of SP-ICP-MS was demonstrated by our research group [40]. The analytical method developed showed an accurate quantification (<5% error) for high functionalized NPTs up to 1 µm. For a fully functionalized NPT, the lower size detectable was reported as 135 nm. More recently, Lai et al. have applied the idea of labelling NPTs with AuNPs with small modifications and improvements [41]. Authors proposed an alternative for the labelling, consisted on the in situ growth of AuNPs at the surface of NPTs, followed by their counting by SP-ICP-MS. In order to remove free AuNPs that could interfere with the quantification, labelled nanoplastics were isolated by Sucrose Density Gradient Centrifugation (SDGC) prior to the analysis by SP-ICP-MS. This approach enabled to enhance the NPT detection limit to 4.6 × 10^8^ NPTs L^−1^ for 269 nm PS NPTs. In addition, the method was applied, after acid digestion and cloud-point extraction, in real environmental waters with high recoveries (72.9–92.8%). Hence, the metal nanoparticles labelling proved to be a promising strategy for nanoplastics detection and quantification by ICP-MS.

## 3. Materials and Methods

### 3.1. Reagents and Standards

Aqueous solutions of cerium, yttrium, thorium, gold, lead, and silver were prepared in ultrapure water from monoelementary standard stock solutions of 1000 mg L^−1^ purchased from SCP Sciences (Courtaboeuf, Villebon-sur-Yvette, France). Silver nitrate (AgNO_3_, ≥99.0), Gold (III) chloride hydrate (HAuCl_4_), lead (II) phthalocyanine, trisodium citrate dihydrate (Na_3_-citrate) and gelatine Type A (300 bloom, isoelectric point IEP ≈ 8) from porcine skin were purchased from Sigma-Aldrich (Saint Quentin Fallavier, France). Standard gold nanoparticle suspensions (AuNPs) with nominal size of 32.7 ± 2 were obtained from LGC Standards (Teddington, London, UK). The synthesis and characteristics of soap-free polystyrene model materials were detailed elsewhere [42]. The synthesis of gelatin coated AuNPS (AuNPS@gel) was described elsewhere [40]. Ultrapure water (18 MΩ cm^−1^) obtained with a MilliQsystem (Millipore, Guyancourt, France) was used throughout.

### 3.2. Sample Preparation Procedures

#### 3.2.1. Labelling with Metal Ions

The PSAA13 labelling is given as an example: equal volumes of PSAA13 and the corresponding metal ionic solution (silver, gold, or lead) were mixed at a ratio of 50:1 in terms of mass concentration. The resulting mixtures were let under stirring overnight and diluted accordingly with ultrapure water in order to have a metal mass concentration lower than 10 µg L^−1^ prior to SP-ICP-MS analysis.

The filtration process was performed with a controlled flow of 0.1 mL min^−1^ using 0.02 µm filters (Whatman Anotop, VWR, Rosny-sous-Bois, France), previously conditioned with 0.5 mL of the ionic solution, and a syringe pump module (NeMESYS, Korbussen, Germany). Prior filtration, equal volumes of PSAA22 and AgNO_3_ were mixed at mass concentration ratios of 100:0.005, 100:0.01, 100:0.05, 100:0.2, 100:0.5; 100:1 and let in contact overnight. The filtrate was diluted accordingly with ultra-pure water in order to have a silver mass concentration lower 10 µg L^−1^ prior to SP-ICP-MS analysis.

#### 3.2.2. Labelling with a Hydrophobic Organometallic Compound

2 mg of lead (II) phthalocyanine was added to a 10 mL suspension of 500 mg L^−1^ of PSAA22 and was left under agitation during 24 h. The resulting mixture was centrifuged 5 min at 4000 rpm and room temperature. The supernatant was recovered, filter through 5 µm PES syringe filters (GE Healthcare, Chalfont Saint Giles, UK), and diluted accordingly with ultra-pure water in order to have a NPT number concentration of around 1 × 10^8^ L^−1^ prior SP-ICP-MS analysis.

#### 3.2.3. Labelling with Metal Nanoparticles

An aliquot of 20 μL of AuNPs@gel suspension was mixed with 20 μL of a water dispersed NPTs (PSAAxx) at a ratio of approximately 1000:1 in terms of particle number. The resulting mixture was centrifugated for 5 min at 4000 rpm. The resulting supernatant was diluted accordingly with ultrapure water in order to have a NPT number concentration of around 1 × 10^8^ part L^−1^ or lower prior to SP-ICP-MS analysis.

### 3.3. Instrumentation

#### 3.3.1. Total Metal Content Analysis

The total silver content was determined by monitoring isotopes ^107^Ag and ^109^Ag with an Agilent 7900 ICPMS (Agilent, Tokyo, Japan). Each sample was analysed by triplicate. Results are expressed as the mean of three measurements.

#### 3.3.2. Single Particle ICP-MS Analysis

Single Particle analysis concerning labelling with metal ions and a hydrophobic organic compound containing a metal ions was carried out with an Agilent 7900 ICPMS equipped with Single Nanoparticle Application Module. The corresponding isotope was monitored with a dwell time of 100 µs during a total acquisition time of 60 s. For the experiments with metal nanoparticles, a NexION 5000 ICP MS with a Syngistix Nano Application Module (Perkin Elmer, Shelton, CT, USA) was used. ^197^Au was monitored with a dwell time of 50 µs during a total acquisition time of 100 s. Transport efficiency was calculated for both instruments according to the particle frequency and particle size methods described by Pace et al. [43] using a standard AuNPs suspension as reference material. The transport efficiency calculated by the particle frequency method was used to determine the NPT number concentration, whereas the transport efficiency calculated by the particle size method was used to determine the mass of Au per NPT particle. The sample flow rate was calculated daily by measuring the mass of water taken up by the peristaltic pump during 2 min. This operation was repeated twice and the average values were used for calculations.

## 4. Conclusions and Future Directions

**Figure 7:**
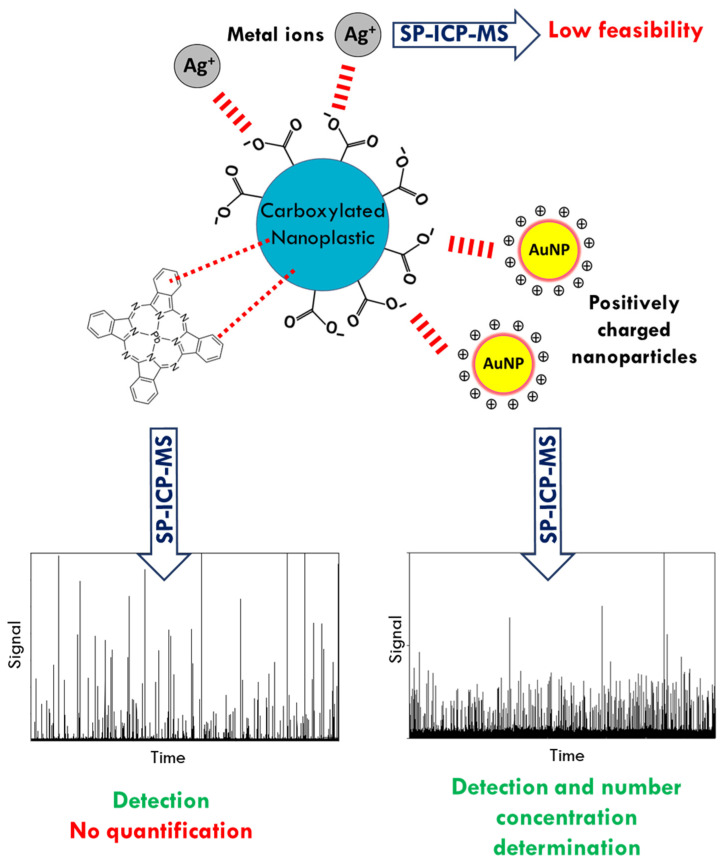
Summary of the different labelling strategies.

In this work, the feasibility of the use of a metal probe for the detection and quantification of NPTs by SP-ICP-MS was tested. Three different labelling based on three metal probes were studied and discussed: metal ions, hydrophobic organometallic compound, and inorganic nanoparticles, which are summarized in Figure 7. The three approaches took advantage of the ability of NPTs to adsorb organic and metal compounds. The labelling through metal ions proved to be of difficult applicability for several reasons, the most important one being the fact that the mass of metal adsorbed onto the NPTs’ surface was not high enough to compensate the background signal produced by metal ions that have not interacted with NPTs. Moreover, the occurrence of desorption processes during the dilution of suspensions prior analysis may not be discarded. On the other hand, the labelling of NPTs was revealed to be much more effective by using hydrophobic organometallic compounds and positively charged gold nanoparticles. The adsorption thanks to the interactions between the phthalocyanine and the phenyl group of the PS combined with the presence of lead in the compound led to the detection of NPTs by the ICP-MS. However, the sample preparation required the removal of the insoluble residue of PbPc before the analysis, which implies a further optimization in order to obtain an accurate quantification of NPTs. Finally, the labelling strategy through positively charged gold nanoparticles has shown promising results both for detection and quantification purposes. The study of NPTs model materials with different number of carboxylic groups per nm^2^ at the surfaces proved that the labelling of gold nanoparticles is dependent on the surface functionalization of NPTs, showing that the adsorption takes place through electrostatic interactions. As a result, while all NPTs studied were detectable through this strategy, the low functionalized ones were not quantified correctly. Further studies should focus on the sample preparation in order to optimize the amount of particles adsorbed onto NPTs.

In conclusion, the labelling with metal probes has shown to be a promising strategy for the sensitive detection of NPTs in aqueous matrices by ICP-MS. In addition, the labelling with metal nanoparticles allowed the quantification of NPTs with a certain degree of functionalization. Finally, the applicability of this latter strategy needs to be further investigated in environmental matrices where natural organic matters and other natural colloids could interfere with the labelling process.

The detection and quantification of NPTs by metal labelling represents a strong potential to obtain not only information about their levels in the environment but also the risks related to their exposure. Thus, this method could find its place in ecotoxicological applications often limited by the available techniques to determine the migration level in different organisms. Also, this new strategy is an interesting opportunity regarding food safety application, especially for water quality and seafood directly exposed to this type of contamination. Finally, this novel tool could be a springboard for the quantification of carbon based nanoparticles in general such as carbon nanotubes with emphasis on potential toxicity [44].

## Figures and Tables

**Table 1:** Samples of labelled PSAA22, silver concentration and PSAA22:Ag ratio.

Sample Name	Silver Concentration mg L^−1^	PSAA22:Ag Ratio
#1	0.005	100:0.005
#2	0.1	100:0.01
#3	0.05	100:0.05
#4	0.2	100:0.2
#5	0.5	100:0.5
#6	1	100:1

**Table 2:** Expected nanoplastic number concentration and nanoplastics number concentration obtained by SP-ICP-MS for different nanoplastic model materials.

NPT	Expected Concentration, NPTs L^−1^	SP-ICP-MS Concentration, NPTs L^−1^
PSAA22	5.58 × 10^7^	6.30 × 10^7^
PSAA18	3.34 × 10^7^	2.48 × 10^7^
PSAA13	5.18 × 10^7^	3.33 × 10^6^
PSAA9	4.16 × 10^8^	2.43 × 10^7^

## Data Availability

The data presented in this study are available in “Nanoplastic labelling with metal probes: analytical strategies for their sensitive detection and quantification by ICP mass spectrometry” and Supplementary Materials.

## Supplementary Materials

The following are available online, Figure S1: Time scans obtained in SP-ICP-MS for PSAA22:Ag at ratio 100:0.2 for (a) 20 000-fold dilution, (b) 1 000-fold dilution.

## Sample Availability

Samples of the compounds are available from the authors.
