# Supplementary material for: Nanoplastic Labelling with Metal Probes: Analytical Strategies for Their Sensitive Detection and Quantification by ICP Mass Spectrometry
Source: Molecules. 2021 Nov 24;26(23):7093. doi: 10.3390/molecules26237093 (PMC8659233; doi:10.3390/molecules26237093)
Supplement: Supplementary file 1 [file molecules-26-07093-s001.zip › molecules-1450724-supplementary.pdf]

Supplementary materials

Nanoplastic Labelling with Metal Probes: Analytical Strategies for Their Sensitive Detection and Quantification by ICP Mass Spectrometry

Lucile Marigliano *, Bruno Grassl, Joanna Szpunar, Stéphanie Reynaud and Javier Jiménez-Lamana

Université de Pau et des Pays de l'Adour, E2S UPPA, CNRS, Institute of Analytical and Physical Chemistry for the Environment and Materials (IPREM), Pau 64053, France
* Correspondence: Lucile.marigliano@univ-pau.fr

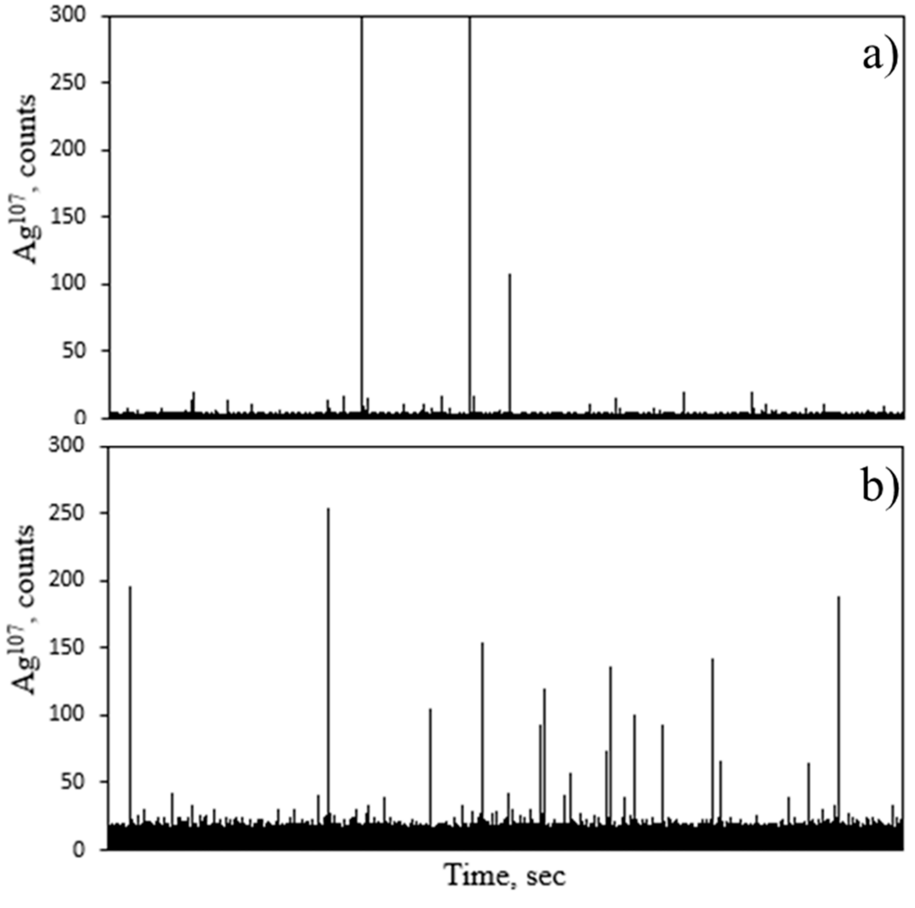

Figure S1. Time scans obtained in SP-ICP-MS for PSAA22:Ag at ratio 100:0.2 for (**a**) 20,000-fold dilution, (**b**) 1000-fold dilution.
